# Improved Flexible Transparent Conductive Electrodes based on Silver Nanowire Networks by a Simple Sunlight Illumination Approach

**DOI:** 10.1038/srep42052

**Published:** 2017-02-07

**Authors:** Pengfei Kou, Liu Yang, Cheng Chang, Sailing He

**Affiliations:** 1 Centre for Optical and Electromagnetic Research, State Key Laboratory of Modern Optical Instrumentation, Zhejiang University, Hangzhou 310058, P. R. China; 2 Department of Electromagnetic Engineering, JORCEP, School of Electrical Engineering, Royal Institute of Technology (KTH), S-100 44 Stockholm, Sweden

## Abstract

Silver nanowire (Ag NW) networks have attracted wide attention as transparent electrodes for emerging flexible optoelectronics. However, the sheet resistance is greatly limited by large wire-to-wire contact resistances. Here, we propose a simple sunlight illumination approach to remarkably improve their electrical conductivity without any significant degradation of the light transmittance. Because the power density is extremely low (0.1 W/cm^2^, 1-Sun), only slight welding between Ag NWs has been observed. Despite this, a sheet resistance of <20 Ω/sq and transmittance of ~87% at wavelength of 550 nm as well as excellent mechanical flexibility have still been achieved for Ag NW networks after sunlight illumination for 1 hour or longer, which are significant upgrades over those of ITO. Slight plasmonic welding together with the associated self-limiting effect has been investigated by numerical simulations and further verified experimentally through varied solar concentrations. Due to the reduced resistance, high-performance transparent film heaters as well as efficient defrosters have been demonstrated, which are superior to the previously-reported Ag NW based film heaters. Since the sunlight is environmentally friendly and easily available, sophisticated or expensive facilities are not necessary. Our findings are particularly meaningful and show enormous potential for outdoor applications.

Transparent electrodes are essential elements in various optoelectronic devices such as light emitting diodes, touch panels, film heaters, and solar cells. This field has been dominated by indium tin oxide (ITO) for several decades due to its high transparency and conductivity^1^. However, the scarcity of indium and the need for high-vacuum deposition and high-temperature annealing make ITO increasingly expensive. Moreover, its inherent brittleness has hindered its potential applications for emerging optoelectronics, which require not only high transparency and conductivity, but also mechanical flexibility^2^,^3^,^4^. Alternatives, such as conducting polymers^5^,^6^, carbon nanotubes^7^,^8^, graphene^9^,^10^,^11^ and metallic networks^12^,^13^, have been proposed recently. Among them, the unstable conducting polymers, with weak conductivity, are still far from the practical implementations^2^; carbon nanotubes are also not competitive due to high junction resistance and their semiconducting properties^3^; despite very high mobility, transparent graphene cannot rival ITO in conductivity because of its low carrier concentration as well as the expensive large-area fabrication processes^1^. In contrast, metallic networks, especially silver nanowire (Ag NW) networks^12^,^13^, which simultaneously possess excellent optical transmittance and electrical conductance, have become well-known as the best replacement to ITO. Instead of expensive fabrication processes for metallic networks with certain patterns, e.g., electron beam lithography, vacuum evaporation/sputtering, etc^14^,^15^,^16^,^17^,^18^,^19^. Ag NWs can be easily synthesized and processed in solution. Therefore, Ag NW networks are very low cost. Flexibility is another important feature, making Ag NW networks increasingly attractive. However, solution-synthesized Ag NWs are always randomly distributed on the substrate, leading to weak wire-to-wire contacts. This issue will become increasingly important for flexible device applications. Besides, polyvinylpyrrolidone (PVP) insulating coatings on the surface of Ag NWs, which originate from the PVP stabilizer typically used to control the nanowire shape and help finely disperse the synthesized nanowires in solution, form barriers for electron transport between wires and give rise to high wire-to-wire contact resistances, which, as a result, further limit the conductivity of the whole network^12^,^20^.

To improve the conductivity of the Ag NW networks, thermal treatment at about 200 °C has been widely employed^20^,^21^,^22^,^23^,^24^,^25^. Combined with mechanical pressing, the temperature can be reduced to be 100–150 °C^26^,^27^,^28^, which can be reduced further with well-controlled humidity^29^. However, these conditions might be detrimental to more sensitive devices, e.g., organic electronics. The wire-to-wire resistance can be reduced by chemical deposition of gold nanoparticles on the surfaces of Ag NWs, which will make them rough, leading to obvious degradation of light transmittance^30^. Other materials (e.g., conducting polymers^31^,^32^, heat-resistant polymer^33^, graphene^28^,^34^,^35^, zinc oxide^36^,^37^, titanium dioxide^38^, or clay nanoplates^39^) have been proposed for hybridization with Ag NWs to enable robust performances in terms of transparency, conductivity, flexibility, etc. Recently, localized welding techniques have attracted much attention. When current is applied to the Ag NW network, localized Joule heating accompanied by electromigration can be generated at the high-resistance Ag NW junctions, allowing good welding of crossed Ag NWs and thus high conductivity^40^. Silver nanoparticles can be selectively grown at the junctions of nanowires to weld the network by a plasmon-induced chemical reaction^41^ or an alcohol-based solution approach^42^. Localized chemical welding can also be realized through capillary condensation^43^. Since Gannet *et al*. reported a light-induced plasmonic nanowelding technique based on the localized and self-limited welding effects^44^, this method has been developed quickly and widely applied as an effective post-treatment of Ag NW networks^45^,^46^,^47^. In those reports, light source with excessively high intensity were used, e.g., a broadband tungsten-halogen lamp with a power density of approximately 30 W/cm^2 ^44, and pulsed xenon lamps^45^ or UV lasers^46^,^47^. Especially for pulsed lasers, the illumination area is limited, resulting in low treatment efficiency. If those sources are replaced by electron beams^48^ or plasma^49^, expensive high-vacuum chambers become necessary.

In contrast, we show in this paper that the Ag NWs fabricated by our previously-reported full-solution polymethylmethacrylate (PMMA)-assisted spin-coating method^50^ can be partially welded under the exposure of natural sunlight with power density of only 0.1 W/cm^2^ (i.e., 1 Sun, 300 times weaker than that reported in ref. (44)). Better optical, electrical and flexibility properties can be obtained for samples after the sunlight illumination than for the as-deposited samples, as well as ITO. Numerical simulations as well as experimental investigations on different solar concentrations are performed to clearly demonstrate the physical mechanisms behind the sunlight-induced improvement of conductivity of the Ag NW networks. Finally, uniform Ag NW networks are explored as a flexible transparent electrode for film heaters. Based on the sunlight-illuminated Ag NW networks, our flexible film heater is able to obtain higher temperatures than previously-reported Ag NW film heaters^33^,^39^,^48^,^51^ when working at the same voltage. This indicates superior effectiveness of natural sunlight illumination over the previous treatments for Ag NW networks, including combination with heat-resistant polymer^33^ and clay nanoplates^39^ as well as electron beam irradiation^48^. Since sunlight is totally environmentally friendly and easily available, we do not need any special post-treatments of the Ag NW network transparent electrodes, let alone any sophisticated or expensive facilities. It is very economical and convenient, and therefore promising for large-scale applications, especially outdoor applications, e.g., automobile-window defrosters, flexible outdoor panel displays, and solar cells, where the Ag NW networks can be incorporated directly without any post-treatment and will be self-improved over the course of natural exposure to natural sunlight.

## Results and Discussion

**Figure 1:**
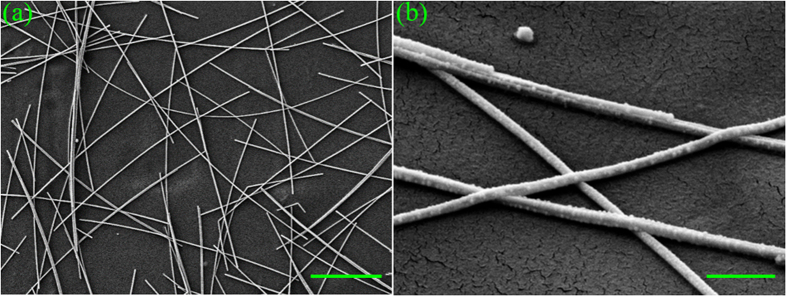
Scanning electron microscope (SEM) images of the as-deposited Ag NW network. (**a**) scale bar: 5 μm; (**b**) scale bar: 500 nm.

**Figure 2:**
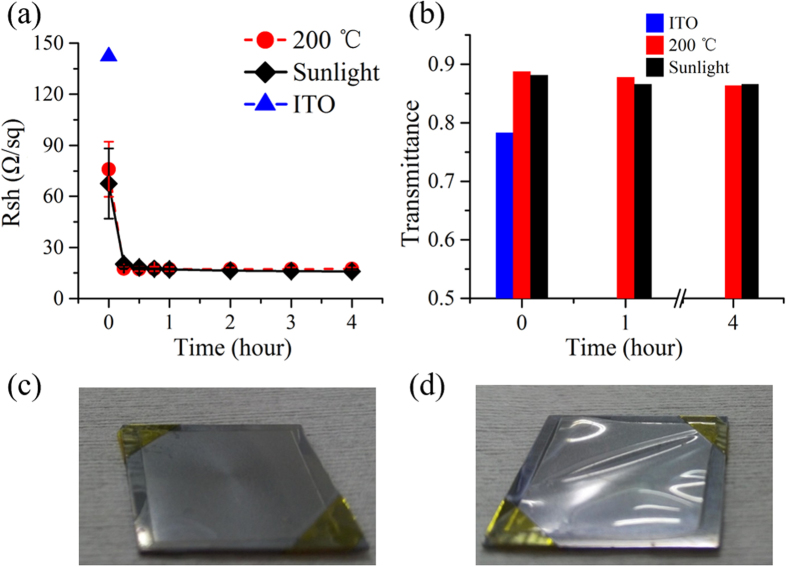
(**a**) Sheet resistance (R_sh_) as a function of treatment time for samples treated with 1-Sun sunlight illumination (black diamonds) and thermal annealing at 200 °C (red circles), respectively. (**b**) Histogram of the light transmittance at 550 nm before and after different periods of time for both treatment methods in (**a**). The values of R_sh_ and light transmittance at wavelength 550 nm for a 571-nm thick ITO electrode are also indicated in Fig. 2a (blue triangle) and 2b (blue), respectively. (**c**) No deformation appeared when the sample was exposed to the sunlight for 1 hour. (**d**) Serious deformation occurred for the Ag NW network on PE after thermal annealing at 200 °C for the same period of time.

The Ag NW networks were prepared on polyethylene (PE) substrates by using our previously-reported full-solution PMMA-assisted spin-coating method^50^. Details are described in Methods. The Ag NWs had lengths of ~15 μm and diameters of ~60 nm. They were randomly distributed on the PE substrate as shown in Fig. 1(a), loosely contacted to each other as shown in Fig. 1(b), and inspected by a scanning electron microscope (SEM; see Methods). The measured sheet resistance (R_sh_) is over 60 Ω/sq with transmittance as high as ~87% at 550 nm, as shown in Fig. 2(a and b). These loose contacts between Ag NWs must be the main reason behind the high R_sh_.

In order to improve the wire-to-wire contacts and thus reduce R_sh_, the as-deposited Ag NW networks were exposed to sunlight in ambient environment. In order to guarantee repeatability and reliability, a standard solar simulator (SAN-EI ELECTRIC XES-40S2-CE) was employed to generate stable sunlight of 0.1 W/cm^2^ (i.e., 1 Sun; until otherwise specified), 300 times lower than that reported in ref. (44), but high enough to improve the junction contacts between Ag NWs. For comparison purposes, conventional thermal annealing was also conducted by heating the samples on a hot plate at 200 °C in ambient environment. Figure 2(a) shows the sheet resistance (R_sh_) variations when time for both sunlight exposure (black diamonds) and heat treatment (red circles) increases. In both cases, R_sh_ drops quickly to ~20 Ω/sq from their initial values (over 60 Ω/sq) after only a 15-min treatment. As time increases, R_sh_ remains almost unvaried for thermally-annealed samples. Interestingly, for the case where the Ag NW network is exposed to the sunlight, R_sh_ continues to decrease at a very slow rate. After a 1-hour treatment, it becomes lower than that seen in the case of thermal annealing. The extremely small error bars mean very good repetition. Since both treatment methods do not seriously degrade the transmission spectra, which are always flat in the visible and near-infrared wavelength range for Ag NW networks, only the values of the light transmittance at 550 nm are demonstrated and plotted in Fig. 2(b) for clarity. It indicates that high transmittance of ~87% @ 550 nm can be kept when the electrical conductivity is improved for both cases. From these results, it is seen that sunlight exposure is equally effective as the conventional thermal treatment, but much gentler than the latter. Serious deformation was observed in the sample heated at 200 °C for only 1 hour (Fig. 2(d)), but nothing changed for the sample under the sunlight illumination for the same period of time (Fig. 2(c)). Therefore, the natural low-intensity sunlight treatment is more advantageous and competitive for wider applications, especially for organic or polymer devices with low thermal budgets. For additional comparison, we prepared ITO transparent electrodes by sputtering at room temperature (Methods). As both R_sh_ and light transmittance increased with the decreasing film thickness, a 571-nm thick ITO film with R_sh_ ~142.2 Ω/sq (Fig. 2(a)) and its transmittance of ~0.78 (Fig. 2(b)) was chosen for comparison. This indicates again the superiority of our transparent electrodes based on the sunlight-treated Ag NW networks in terms of both R_sh_ and transmittance.

**Figure 3:**
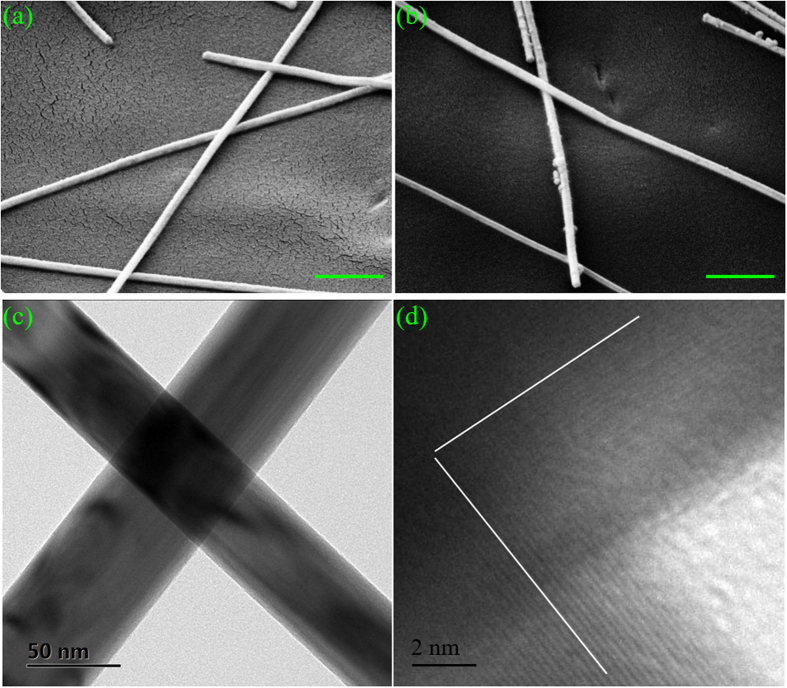
45°-tilted SEM images of the Ag NW networks after (**a**) 1-hour and (**b**) 4-hour sunlight illumination with solar concentration of 1 Sun. Both scale bars in the SEM images are 500 nm. (**c**) Low-magnification and (**d**) high-resolution TEM images of a representative Ag NW junction illuminated by the 1-Sun sunlight for 1 hour.

Figure 3(a and b) show SEM images of the Ag NW networks after 1- and 4-hour treatments of sunlight illumination with solar concentration of 1 Sun, respectively. Compared to the SEM images of the as-deposited Ag NW network shown in Fig. 1, the Ag NWs themselves did not change, but the junctions between them were welded after 1-hour exposure to the sunlight, though the welding was very subtle and slight (Fig. 3(a)). Such effects were similarly induced by the plasmonic welding previously reported in ref. (44). However, due to the 300 times lower intensity of the sunlight (0.1 W/cm^2^) compared to that of the tungsten halogen lamp (30 W/cm^2^)^44^, we observed a much smaller degree of soldering instead of full welding. Both transmission electron microscope (TEM) images (Fig. 3(c and d)) and selected area electron diffraction patterns (Supporting Information, Fig. S1) showed that pentagonally twinned crystal structures of the crossed Ag NWs were not interrupted by the junction, indicating extremely inconspicuous welding. When the exposure time increased to 4 hours, there were still no obvious morphological variations as shown in Fig. 3(b). Therefore, R_sh_ did not show much more significant improvement after 1-hour sunlight illumination, as shown in Fig. 2(a). In comparison with the sunlight illumination approach, the 1- and 4-hour thermal annealing treatments had more significant effects, resulting in a higher degree of welding between Ag NWs as shown in Fig. S2A and S2B, respectively. Despite the slight welding effects, the low-intensity sunlight illumination is still sufficient for very good conductivity of Ag NW networks, as shown in Fig. 2, as well as excellent flexibility, to be demonstrated later. Because the Ag NW junctions were only slightly soldered, the sample surface roughness measured by atomic force microscopy (Supporting Information, Fig. S3) was marginally reduced, with the averaged values of root mean square (RMS) ranging from 114.0 nm for the as-deposited sample to 106.1 nm and 105.6 nm when the sample was exposed to the sunlight for 1- and 4-hours, respectively.

**Figure 4:**
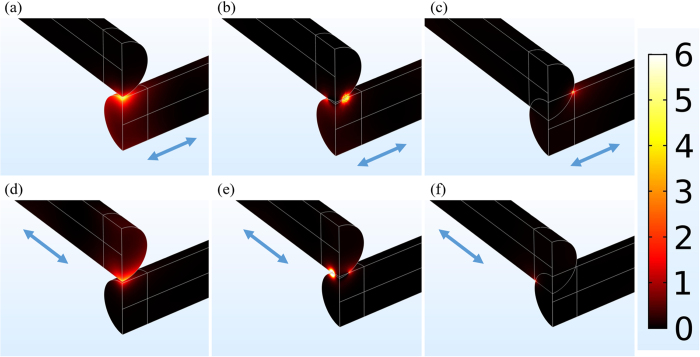
Simulated heat generation profiles at a junction formed by two orthogonal Ag NWs with (**a**,**d**) a 2-nm gap, (**b**,**e**) a 2-nm overlap (just interpenetrating) and (**c**,**f**) a 20-nm overlap, illuminated by a plane wave with a wavelength of 510 nm. The light polarization is indicated by blue arrows, either parallel (top: **a**–**c**) or perpendicular (bottom: **d**–**f**) to the bottom Ag NW.

To illustrate the tiny welding induced by the sunlight illumination, numerical simulations were performed based on a finite-element method (Comsol Multiphysics 5.2). Details are described in Methods. Figure 4 shows the simulated heat generation profiles for the light polarization parallel or perpendicular to the bottom Ag NW in an orthogonally crossed Ag NW junction, when the two wires are separated by a 2-nm air gap, estimated by the 1-nm thick PVP coatings on the Ag NW surfaces, are just interpenetrated with a 2-nm overlap, and are more overlapped with a 20-nm interpenetration area, respectively. From Fig. 4, it is seen that the bottom Ag NW gains more heat than the top Ag NW when the polarization is parallel to it for all the cases and vice versa. For both polarizations, the heat generation at the junction strongly depends on the gap/overlap size. Actually, upon sunlight exposure, strongly localized optical fields are excited at the wire-to-wire junctions separated by the 1-nm thick surface PVP coatings, where heat is generated by the non-radiative decay of localized surface plasmons (as demonstrated in Fig. 4(a and d)). As the gap becomes smaller, more heat is produced with stronger optical field confinement. As a consequence, the insulating PVP coatings are removed by the accumulated heat and at the same time, the crossed Ag NWs become in touch and then melted together, leading to quickly dropping R_sh_ (Fig. 2(a)). As the melting degree increases, the localized surface plasmons move away from the junction and the intensity becomes weaker (as demonstrated in Fig. 4(b,e and c,f)). In this case, heat generation becomes lower and further soldering is limited. On the other hand, when a certain melting degree occurs, the junction resistance becomes insensitive to it and starts not to dominate R_sh_ because the current can flow freely through the junction. The combined actions contribute to the almost unvaried R_sh_ as the treatment time increases (Fig. 2(a)). Based on this analysis, it was also easy to understand the behavior of R_sh_ for the thermally annealed sample (Fig. 2(a)), which experienced similar welding process after PVP removal, though the optical self-limiting effect could not apply to it. After 1-hour treatment, the soldering became increasingly significant but did not play a key role in influencing R_sh_. That is, a higher degree of soldering did not mean a lower R_sh_ (Fig. 2(a)). This is also the reason for the sample illuminated by the 1-Sun sunlight for over 1 hour with a lower welding degree to have a lower R_sh_ than the thermally annealed sample.

**Figure 5:**
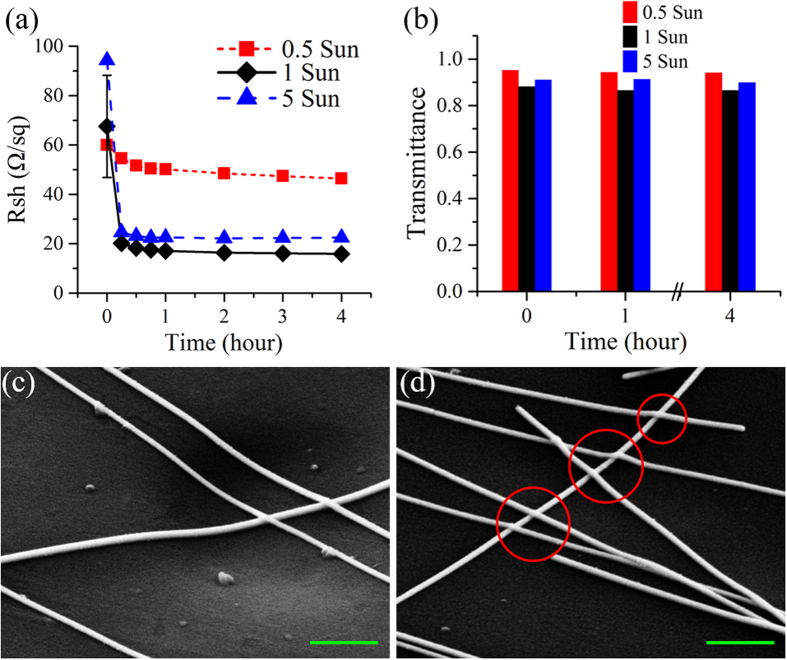
(**a**) Sheet resistance (R_sh_) as a function of treatment time for sunlight-illuminated samples with different power densities (red squares: 0.5 Sun; black diamonds: 1 Sun; blue triangles: 5 Sun), respectively. (**b**) Histogram of the light transmittance at 550 nm before and after different periods of time for all the treatments in (**a**). 45°-tilted SEM images of the samples after 4-hour sunlight illumination with concentration of (**c**) 0.5 Sun and (**d**) 5 Sun, respectively. Red circles indicate apparent welding between Ag NWs. Both scale bars are 500 nm.

To further elaborate and verify the above theoretical analysis, we investigated the effect of sunlight concentration on the performance of the Ag NW networks. The behaviors of both R_sh_ and light transmittance in the cases under 0.5-Sun (0.05 W/cm^2^, minimal value achievable in our laboratory) and 5-Sun (0.5 W/cm^2^, maximal value achievable) sunlight illumination were demonstrated in Fig. 5(a and b), respectively, in comparison with the case treated by the 1-Sun (0.1 W/cm^2^) sunlight exposure. As the illumination time increases, no obvious degradation is observed in light transmittance (Fig. 5(b)), and meanwhile, improvement of R_sh_ is achieved (Fig. 5(a)) for all the solar concentrations (even as low as 0.5 Sun). This means that the natural sunlight with varied power intensities will not degrade but still be able to improve the performance of the Ag NW networks. From Fig. 5(a), it is also seen that as the solar power increases, R_sh_ drops faster. After 15 min illumination, the R_sh_ values of the 5-Sun and 1-Sun illuminated samples were improved by 73.9% and 70.2%, respectively, while R_sh_ of the 0.5-Sun illuminated sample was only reduced by 9%. Besides, the treatment time necessary for R_sh_ to become stable was also shorter with stronger irradiation (45 min for the 5-Sun sample, 2 hours for the 1-Sun sample, more than 4 hours for the 0.5-Sun sample whose R_sh_ continued decreasing during the whole treatment process). With even higher power density, e.g., 30 W/cm^2^ (equal to 300 Sun) from a broadband tungsten-halogen lamp^44^, the treatment time could be further reduced, e.g., to 60 s^44^. The SEM images (Figs. 3(a), 5(c and d)) indicate that the crossed Ag NWs illuminated by the 0.5-Sun, 1-Sun and 5-Sun sunlight experienced similar morphology changes (including surface PVP removal and the simultaneous melting between Ag NWs in touch) to those treated by 300-Sun illumination reported in ref. (44). If the crossed Ag NWs simply keep in touch without being soldered, the significant decrease of R_sh_ (over 70%) cannot be observed for the samples treated by the sunlight with the concentration over 0.5 Sun (Fig. 5(a)). The increasingly significant soldering follows the rising solar concentration, confirming again the important role of the localized surface plasmons confined at the junctions in welding of Ag NWs. Note that the solar concentration of 1 Sun seems strong enough to obtain the optimal performance of the Ag NW networks, because the improvement of R_sh_ was almost the same for the 1-Sun (76.5%) and 5-Sun (76.2%) exposed samples after 4-hour treatment compared to 22.6% for the 0.5-Sun sample. Because of the easy availability, the effective 1-Sun sunlight treatment was employed in the following experiments.

**Figure 6:**
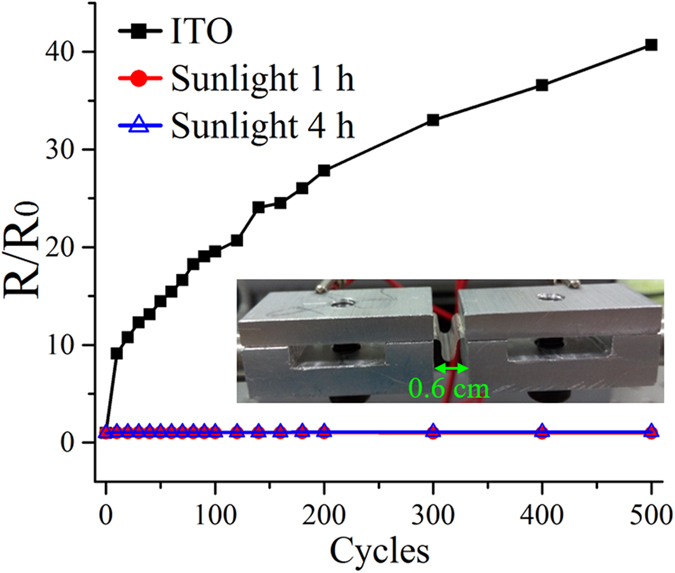
Normalized resistance as a function of the number of bending cycles of the transparent film heaters based on the Ag NW network transparent electrodes on PE after being exposed to sunlight for 1 hour (red circles) and 4 hours (blue triangles), compared to that of the ITO electrode on PE (black squares). The inset shows the setup of the bending test, where the transparent electrode is fixed and bent to a minimal curvature of <0.15 cm in radius.

In order to explore the potential of the sunlight-illuminated Ag NW networks for transparent film heaters, we first examined the mechanical flexibility of our transparent electrodes through bending tests (Methods). Even though our spin-coating method and sunlight illumination treatment allow large-area fabrication of flexible Ag NW network transparent electrodes^50^, only small-size film heaters (~1.8 cm × 1.8 cm) are demonstrated. The transparent film heaters were fixed and bent to a minimal curvature radius of <0.15 cm via the moving aluminium supports (the inset of Fig. 6). For each bending cycle, the resistance, R, was recorded by a multimeter through the conductive aluminium supports connecting to the silver paste contacts on the opposite edges of the electrodes. The initial resistances (R_0_) were ~93.4 Ω and ~31.7 Ω for our Ag NW networks after 1- and 4-hour sunlight treatments, respectively. For comparison purposes, a 571-nm thick ITO electrode on PE with R_0_ ~106.8 Ω was also prepared and examined under the same bending test conditions. Figure 6 shows normalized resistance to R_0_ versus the number of bending cycles. From this figure, it is seen that, for ITO, R continues to rise as the number of bending cycles increases, while the values of R for the two Ag NW network transparent electrodes remain almost unvaried, even when they are bent 500 times. This indicates the superior durability of our flexible electrodes compared to ITO even though they are illuminated by the 1-Sun sunlight for only 1 hour.

**Figure 7:**
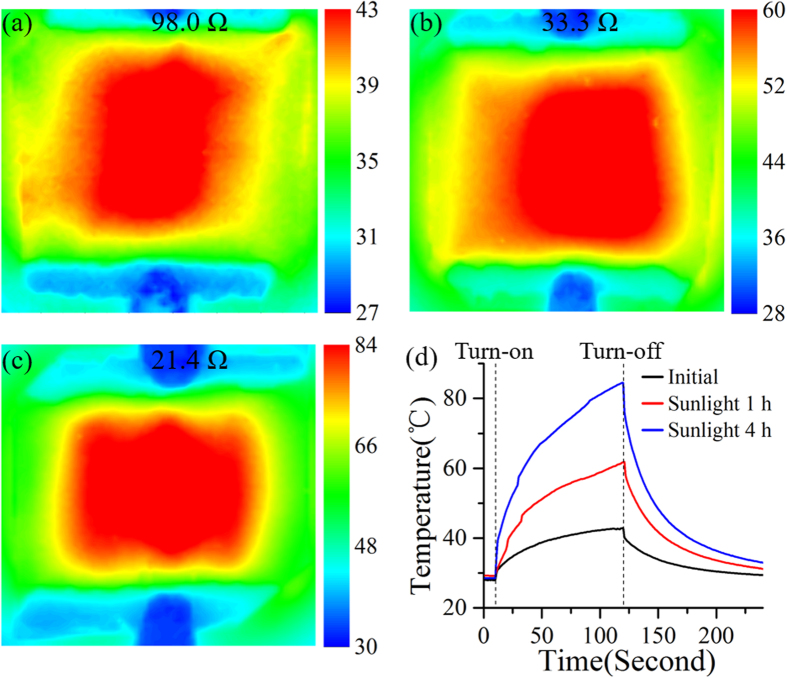
Infrared images of flexible transparent film heaters based on (**a**) the as-deposited Ag NW network, and Ag NW networks irradiated by sunlight for (**b**) 1 hour and (**c**) 4 hours, respectively. (**d**) Time-dependent central temperature of the heaters based on the as-deposited Ag NW network (black), and the Ag NW networks irradiated by sunlight for 1 hour (red) and 4 hours (blue), respectively.

When supplied with a constant DC (direct current) voltage through the silver paste edge contacts of the film heaters, the intermediate Ag NW networks can be heated, and higher heat dissipation can be obtained for heaters with smaller R values. Figure 7(a–c) showed the temperature profiles recorded by an infrared thermometer for the as-deposited sample and the samples after 1- and 4-hour 1-Sun sunlight illumination, respectively, when the same DC voltage of 5 V was applied for 120 s. Compared to the as-deposited sample without any post-treatments (Fig. 7(a)), the heater exposed to sunlight for 1 hour was able to achieve a higher and more uniform heat distribution over the whole area of the Ag NW network (Fig. 7(b)) due to the lower R = 33.3 Ω (versus 98.0 Ω for the as-deposited sample shown in Fig. 7(a)). For the 4-hour sunlight-exposed sample with the lowest R, the highest and most uniform temperature profile was obtained (Fig. 7(c)). Time-dependent temperatures at the center of the three samples were plotted in Fig. 7(d), further indicating notable conductivity improvement of the sunlight exposure. The three curves followed similar trends as previous results^33^,^39^,^48^,^51^ when the bias was turned on and turned off. The sample with 4-hour sunlight illumination could reach a maximum temperature of ~84 °C, which was much higher than that achieved by the as-deposited Ag NW film heater^51^ and that achieved by combining Ag NWs with clay nanoplates^39^ and heat-resistant polymer (with comparable transmittance and sheet resistance)^33^ at the same 5 V bias. This temperature obtained at 5 V was also higher than that of the sample irradiated by electron beam at 7 V^48^. It is noteworthy that the turn-on time in our work was not long enough for the temperature to become stable, and the maximal temperature should be much higher than 84 °C. As a result, we can conclude that our sunlight exposure is superior to the previous treatments^33^,^39^,^48^. From Fig. 7(d), it was also seen that the rate of increase of temperature was also higher for the sample with lower R due to the higher current passing through the heater, which generated a more significant Joule heating effect and, thereby, the simultaneous greater reduction of the wire-to-wire contact resistances.

**Figure 8:**
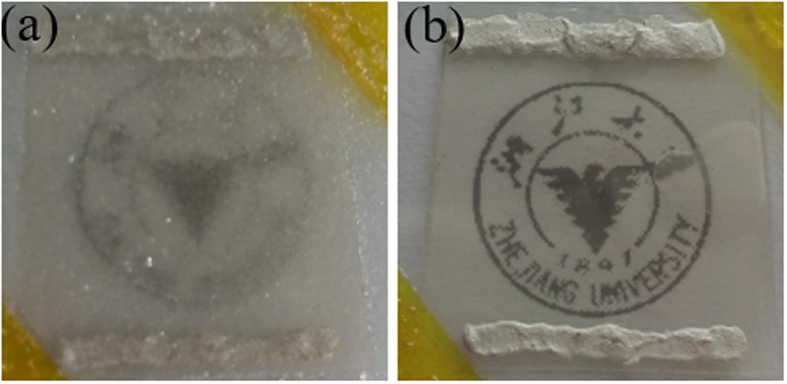
(**a**) Defrosting test of our transparent film heater based on the 4-hour sunlight-exposed Ag NW networks: (**a**) after frost formation; (**b**) after application of 5-V DC voltage for 90 s.

Finally, a defrosting test was conducted to demonstrate the potential of our flexible transparent film heater as an efficient defroster. For operating convenience, the heater based on the 4-hour sunlight exposure was attached to a piece of glass and put into a refrigerator until frost formed and transparency diminished (Fig. 8(a)). After the heater was supplied with a voltage of 5 V for as short as 90 s, the frost was removed completely, and transparency was recovered (Fig. 8(b)). We noticed that the resistance between the two silver side contacts remained almost unvaried before and after the defrosting test (only 0.048 times larger than before).

## Conclusion

To summarize, we have demonstrated a promising method based on sunlight irradiation to remarkably improve the electrical conductivity of Ag NW networks without any significant degradation of the light transmittance. Since the power density is very low (0.1 W/cm^2^, 1 Sun), 300 times weaker than that reported in ref. (44), only slight welding between Ag NWs has been observed. Despite this, R_sh_ values of less than 20 Ω/sq, transmittance values of ~87% at 550 nm, and excellent flexibility have still been achieved for Ag NW networks after sunlight illumination for 1 hour or longer, all of which are far superior to ITO. The physical mechanism has been systematically investigated through numerical simulations and the experiments on varied solar concentrations. Due to the reduced resistance of Ag NW networks after 1-Sun sunlight treatment, high-performance transparent film heaters as well as efficient defrosters have been demonstrated, which are superior to the previously-reported film heaters based on Ag NWs hybridized with clay nanoplates^39^ and heat-resistant polymer^33^ or treated by electron beam irradiation^48^, let alone the film heaters based on as-deposited Ag NWs^51^. Furthermore, sunlight is both environmentally friendly and easily available. Therefore, sophisticated and expensive facilities are not necessary to apply specific post-treatments to Ag NW network transparent electrodes. It is very economical and convenient. Our findings are particularly meaningful and inspiring for outdoor applications, e.g., automobile-window defrosters, flexible outdoor panel displays, and solar cells, where the Ag NW networks directly installed will be self-improved later during exposure to the natural sunlight, even with the solar power density as low as 0.5 Sun.

## Methods

### Preparation of Ag NW Networks

Ag NWs (length: ~15 μm, diameter: ~60 nm) were purchased from Blue Nano and deposited on polyethylene (PE) by our previously-reported polymethylmethacrylate (PMMA)-assisted spin-coating method^50^. Briefly, the as-purchased Ag NWs, suspended in ethanol (5 mg/mL), were first concentrated to 40 mg/mL and then mixed with PMMA 679.04 at a volume ratio of 1:4. After sufficiently mixed by a mixer, the suspension was spin-coated onto a PE substrate that had been carefully cleaned and attached to a Si substrate first at 2000 rpm for 3 s and then at 4000 rpm for 59 s. In order to remove PMMA, we immersed the suspension-coated substrate in acetone for 2 hours, which was long enough for the PMMA to be completely dissolved. During this time, the PMMA gradually dissolved, and the Ag NWs originally embedded in it fell on top of the substrate, forming a random nanowire network. After being removed from the solution, the sample with the Ag NW network was dried in air. Then, the samples were exposed to sunlight of 0.1 W/cm^2^ (1 Sun) generated by a standard solar simulator (SAN-EI ELECTRIC XES-40S2-CE) in ambient environment. The sunlight with a lower power density of 0.05 W/cm^2^ (0.5 Sun; the minimal value achievable) was easily obtained, while a higher power density of 0.5 W/cm^2^ (5 Sun; the maximal value achievable) was produced by concentrating the sunlight with a convex lens. For comparison, conventional heat treatments were also conducted by heating the samples on a hot plate at 200 °C in ambient environment. For further comparison, ITO transparent electrodes were prepared by RF sputtering (Kurt J Lesker PVD75; power: 80 W, pressure: 3 mTorr; room temperature).

### Ag NW-Based flexible transparent Film Heaters

Flexible transparent film heaters (18 mm × 18 mm) were made by applying silver conductive paste to the Ag NW networks on PE in a two-terminal side-contact configuration. Bending tests were conducted by using a home-made apparatus in which the film heaters were fixed and bent to a minimal curvature radius of <0.15 cm by moving aluminum supports (the inset of Fig. 6). The film resistance after each bending cycle was recorded by a multimeter through the conductive aluminum supports connecting to the silver paste contacts on the electrodes. For the heating demonstration, the DC voltage was supplied to the film heater through the edge silver paste contacts by a source meter (KEITHLEY 2450). The temperature distributions were recorded by an infrared thermometer (Mikron Infrared M7500). For the defrosting test, the Ag NW network on PE was attached to a glass substrate and then put in a refrigerator until frost formed. Later, a 5-V DC voltage was applied to the film heater to remove its frost and regain transparency.

### Characterization

The morphology of the Ag NW networks was inspected with a Carl Zeiss Utral 55 field emission scanning electron microscope. In order to avoid charging of the insulating PE substrate and to improve the image clarity, an ultrathin film of gold was sputtered onto the samples. However, the gold film was too thin to be a continuous film, making the Ag NWs appear very rough. Based on our previous experience^50^, the PMMA carrying the Ag NWs is removed completely by acetone, and nothing remained on the Ag NW surfaces. Therefore, we inferred that the small particles on the Ag NW surfaces should be gold. Fortunately, it did not affect the inspection of the wire-to-wire junctions. High-resolution transmission electron microscope (HR-TEM) and selected area electron diffraction (SAED) were observed by JEOL JEM-2100 to characterize the junctions of Ag NWs. Atomic force microscope (AFM) images were taken by SII SPI-3800N. The sheet resistances were measured by our home-built four-probe measurement system. The transmission spectra were measured by a spectrometer based on an integrating sphere and normalized to that of a pure PE film.

### Simulation

A finite-element method was employed to conduct three-dimensional numerical simulations of the optical behaviors of two orthogonally crossed Ag NWs surrounded by air (Comsol Multiphysics 5.2). In the model, the Ag NW diameters were set to 60 nm according to the Ag NWs used in the experiments, but their lengths were set to 600 nm (much shorter than the actual length) to save memory of the computer. The initial gap between the Ag NWs before sunlight exposure was 2 nm, estimated by the ligand spacing of the surface PVP. After welding, the gap disappears and overlapping appears with negative values for gap (e.g., a −2 nm gap means a 2-nm overlap). A plane wave illuminated normally from the top. Considering that there were many different situations for crossed Ag NWs in a random network during the melting process and the localized surface plasmons could be excited by light with many different wavelengths, the light wavelength was set (but not limited) to 510 nm without loss of generality. In order to simplify the model, perfect electric conductor and perfect magnetic conductor boundaries were treated along the directions perpendicular and parallel to the light polarization, respectively. In this case, only a quarter of the junction was necessary for the simulation. With this model, heat generation profiles, i.e., light absorption distributions, were obtained by the electric intensity multiplied by the imaginary parts of the dielectric constants of Ag^52^.

## Additional Information

**How to cite this article**: Kou, P. *et al*. Improved Flexible Transparent Conductive Electrodes based on Silver Nanowire Networks by a Simple Sunlight Illumination Approach. *Sci. Rep.* **7**, 42052; doi: 10.1038/srep42052 (2017).

**Publisher's note:** Springer Nature remains neutral with regard to jurisdictional claims in published maps and institutional affiliations.

## Supplementary Material

Supplementary Information
