# Supplementary material for: Improved Flexible Transparent Conductive Electrodes based on Silver Nanowire Networks by a Simple Sunlight Illumination Approach
Source: Sci Rep. 2017 Feb 7;7:42052. doi: 10.1038/srep42052 (PMC5294563; doi:10.1038/srep42052)
Supplement: Supplementary Information [file srep42052-s1.doc]

Supporting Information

Improved Flexible Transparent Conductive Electrodes based on Silver Nanowire Networks by a Simple Sunlight Illumination Approach

Pengfei Kou, Liu Yang* Cheng Chang, and Sailing He*


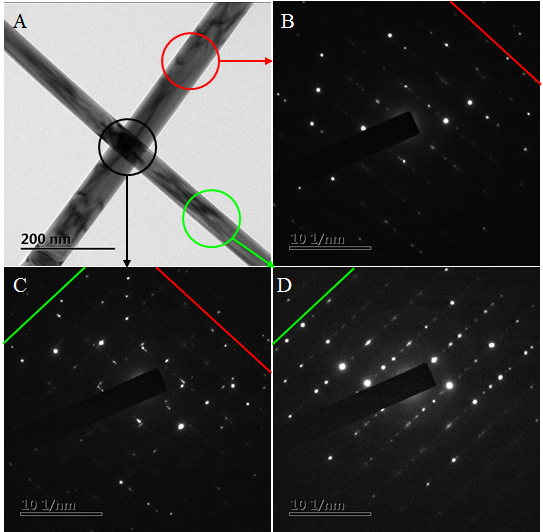


**Figure S1** Selected area electron diffraction (SAED) of a representative Ag NW junction after 1-hour sunlight illumination. A) Low-magnification TEM image of the Ag NW junction. The circles represent the approximate size and location of the diffraction apertures used for the SAED patterns in B-D. B) SAED pattern of the bottom Ag NW from the junction. C) SAED pattern of the junction. D) SAED pattern of the top Ag NW from the junction.

From the above selected area electron diffraction (SAED) patterns of the crossed Ag NWs, it was seen that the strong primary diffraction spots, as well as the relatively weaker double spots induced by the pentagonally twinned crystals, formed parallel lines of spots all along the red line in Figure S1B for the bottom Ag NW and the green line in Figure S1D for the top Ag NW, respectively. At the junction, there were double diffraction spots along the two directions with roughly equal intensity, leading to a grid pattern (Figure S1C). This is different from the fully-welded Ag NW junctions induced by electron beam illumination [48] or high-intensity (30 W/cm^2^) tungsten halogen lamps [44]. Therefore, no obvious welding was visible for our sample under 1-hour sunlight exposure.


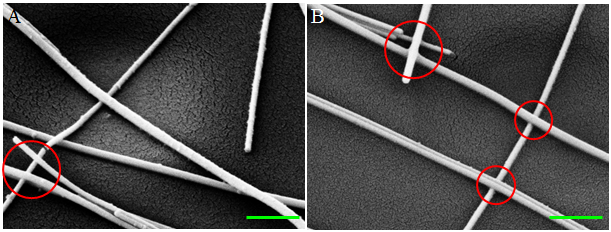


**Figure S2.** 45°-tilted SEM images of the Ag NW networks after A) 1-hour and B) 4-hour thermal annealing at 200 °C. Red circles indicate apparent welding between Ag NWs. Both scale bars are 500 nm.


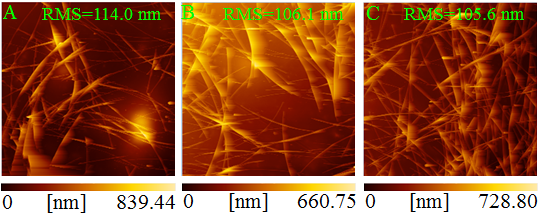


**Figure S3**. AFM images of A) the as-deposited Ag NW network and the Ag NW networks after being illuminated by the sunlight for B) 1 hour and C) 4 hours.
